# 

**Published:** 1853-07

**Authors:** 


### THE WEST.

EDITED BY

JAMES TAYLOR, M. D., D. D. S.

PUBLISHED QUARTERLY.
AT $2 PER ANNUM.

AGENTS.

J. B. DUNLEVY, Pittsburgh, Pa.
Drs. SPALDING & FITHIAN, St. Louis, Mo.
Messrs. JONES, WHITE & McCURDY, Phila. New York City, and Boston, Mass.
Dr. J. M. BROWN, Cincinnati.
SOLOMON BROWN, New York city.
JOHN KLEIN, Phil. Pa.

CINCINNATI:
PRINTED BY T. WRIGHTSON, 12 WEST SECOND STREET.
1853.

